# HIV-ASSOCIATED NON HODGKIN LYMPHOMA: A CASE SERIES STUDY FROM TURKEY

**DOI:** 10.21010/ajid.v14i2.7

**Published:** 2020-07-31

**Authors:** Kayra Somay, Sidar Çöpür, Emre Osmanbaşoğlu, Helin Masyan, Harun Arslan, Olga Meltem Akay, Süda Tekin, Burhan Ferhanoğlu

**Affiliations:** 1 Internal Medicine Department, Koç University Medical School, İstanbul, Turkey; 2 Koç University Medical School, İstanbul, Turkey; 3 Hematology Department, Koç University Hospital, İstanbul, Turkey; 4 Internal Medicine Department, Koç University Hospital, İstanbul, Turkey; 5 Hematology Department, Koç University Medical School, Istanbul, Turkey; 6 Infectious Diseases and Clinical Microbiology Department, Koç University Medical School, Istanbul, Turkey

**Keywords:** Human immunodeficiency virus, lymphoma, non-Hodgkin lymphoma, anti-retroviral therapy

## Abstract

**Background:**

Human immunodeficiency virus (HIV) is a global health concern with major risks for opportunistic infections and predisposition to malignancies including Kaposi sarcoma associated with Human Herpes Virus-8 (HHV-8) and non-Hodgkin lymphoma (NHL) commonly associated with Epstein Barr Virus (EBV). Although the exact mechanisms of predisposition to certain malignancies are unclear, HIV (+) cancer patients typically have poorer prognosis.

**Materials and Methods:**

We included all five HIV positive NHL patients receiving antiretroviral therapy (ART) and chemotherapy in our clinic and aim to determine their follow-up outcomes associated with ART.

**Results:**

The use of ART in conjunction with chemotherapy regimens lead to better therapeutic outcome in our cases with no mortality over three years of follow-up despite high rates of poor prognostic factors and studies demonstrating 1-year survival rates of approximately 30% in HIV-associated lymphoma. No significant adverse effect has been recorded.

**Conclusion:**

We recommend use of ART along with chemotherapy regimens in HIV positive lymphoma patients for better treatment response.

## Introduction

Human immunodeficiency virus (HIV) is a predisposing factor for certain malignancies including lymphoma. Despite high incidence of concomitant infections with Human Herpes Virus-8 (HHV-8) and Epstein Barr Virus (EBV), primary mechanism underlying malignant transformation is thought to be HIV-related immunosuppression (Grulich *et al.*, 2007). Possible hypothesis include increased apoptotic rates in lymphocytes, defects at natural killer cells, hypergammaglobulinemia and over-proliferation of T-cells at lymph nodes (Haas *et al.*, 2011). In addition to being a predisposing factor for malignancy, HIV-infection is a poor prognostic factor after malignant transformation in terms of mortality and morbidity (Ji and Lu 2017). HIV infection is related to complex karyotypes and leukemic presentation both of which are independent poor prognostic factors, a retrospective study conducted with forty-nine HIV positive Burkitt lymphoma patients (Opie *et al.*, 2020). However, HIV viral load was not associated with mortality in a study conducted with eighty-six participants in Sub-Saharan Africa (Painschab *et al.*, 2019). 25-40% of HIV-infected population has shown to develop malignancy in their life-time while Kaposi sarcoma, cervical cancer and non-Hodgkin lymphoma (NHL) are the most common HIV-associated malignancies. 20% of HIV-infected patients develop NHL during life-time, while 2-3% of those are diagnosed with NHL at the time of HIV diagnosis (Grulich *et al.*, 2007; Rabkin and Yellin 1994). Most predominant subtype of NHL is diffuse large B-cell lymphoma (DLBCL) composing 75% of the cases followed by Burkitt lymphoma (Rabkin and Yellin 1994). NHL incidence was 25-100 fold higher in HIV-infected population before the use of ART, while malignancies remain the most common cause of mortality in developed countries despite the use of ART (Smith *et al.*, 2014). In this study we report five cases of HIV-infected patients developing lymphoma at our clinic over the last 3 years treatment, who received ART in addition to chemotherapy regimens.

### Case 1

A 77-year-old male patient presented to our clinic with multiple palpable cervical, axillary, mediastinal and abdominal lymph nodes. Computed tomography studies revealed multiple abdominal and thoracic lymph nodes (largest being 18.0×9.5 mm at left mediastinum) and splenic infiltrative lesion at 31×32 mm size. Excisional lymph node biopsy specimen was suggestive of DLBCL. The clinical stage was III and the patient was started on combination chemotherapy with rituximab, cyclophosphamide, doxorubicin, vincristine, prednisolone (R-CHOP) and involved field radiotherapy (17 cycles with total of 30.6 Gray). Bone marrow biopsy was not suggestive of lymphoma. The disease recurred after 3 years in which the patient presented with progressive cervical lymphadenomegaly. Meanwhile, the patient was positive for anti-HIV test. Anti-viral therapy and simultaneous chemotherapy with rituximab and bendamustine regimen due to the comorbidities of patient were initiated (Table 1). Due to lack of efficient chemotherapeutic response, chemotherapy regimen was switched to gemcitabine, dexamethasone, cisplatin (GDP) for which patient developed intolerance. Lenalidomide was administered which was then switched to rituximab, cisplatin, cytosine arabinoside, dexamethasone (R-DHAP) and involved field radiotherapy due to lack of adequate therapeutic response. Complete metabolic response was obtained following 4 cycles of R-DHAP and radiotherapy.

### Case 2

A 30-year-old male patient presented to our clinic with the diagnosis of high grade diffuse large B-cell lymphoma/Burkitt lymphoma after excisional biopsy of cervical lymphadenomegaly. He was found to be HIV positive during initial laboratory work-up. Bone marrow biopsy showed no lymphoma involvement. Positron emission tomography (PET)-CT scan revealed left cervical, bilateral axillary, lymph nodes located inferior to left parathyroid gland and bilateral nasopharyngeal involvement. The clinical stage was II and the patient was started on combination chemotherapy with R-CHOP simultaneously with the anti-viral therapy (Table 1). Follow-up of patient has been uneventful after complete metabolic remission detected on PET-CT after six cycles of R-CHOP.

### Case 3

**Figure 1:**
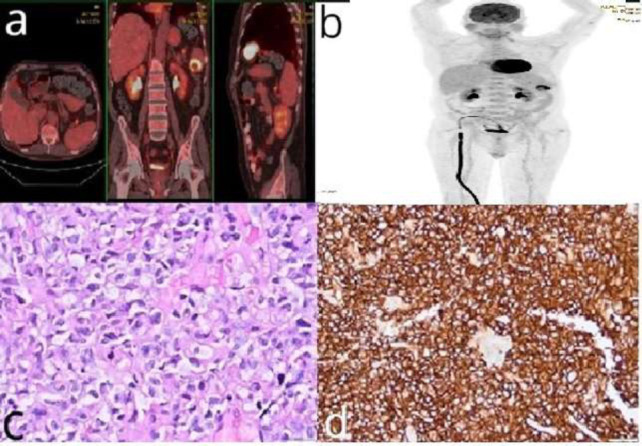
PET-CT of the case 3 demonstrates bilateral thalamus and basal ganglion lesion and splenic lesions with high fluorodeoxyglucose (FDG) uptake (1a/b) while biopsy samples of the same patient demonstrate centroblastic lymphoid cells on hematoxylin & eosin staining (40x magnification) (1c) and tumor infiltration on CD20 staining (200x magnification) (1d).

A 63-year-old male patient presented to internal medicine policlinic with a year history of fatigue, fever and dyspnea on exertion. HIV RNA test previously performed in another center was positive. The patient had been followed up and anti-viral therapy was initiated (Table 1). PET scan (Fig 1a-b) performed upon persistent fever on 2-month follow-up revealed infiltrative lesions on spleen, a lesion with 3 cm diameter (SUVmax=13.5) and another lesion with 75 mm diameter (SUVmax=5.0), for which tru-cut biopsy was obtained, and bilateral lesions at thalamus and basal ganglion (SUVmax=7.8). Pathological examination of specimen indicated DLBCL (Fig 1c-d). Patient developed headache for which brain MRI was planned revealing involvement of thalamus, whereas, cerebrospinal fluid was free of pathology. Patient had received 6 cycles of R-CHOP regimen and intrathecal methotrexate/cytarabine chemotherapy after being considered as stage IV DLBCL. Treatment led to complete metabolic response of cervical lymph nodes and partial metabolic regression of spleen. Follow-up of the patient has been uneventful.

**Table 1:** General characteristics of cases with HIV-associated lymphoma in our clinic

CASE	AGE	AGE AT DIAGNOSIS	CD4 CELL COUNT	LDH Levels	HIV RNA	ANTİ-RETROVIRAL TREATMENT	LYMPHOMA TYPE	PATHOLO GY	LYMPHOMA TREATMENT	IPI / STAGE / ECOG
1	77	73	Initial: 129 cells/ μL 6 months after treatment: 228 cells/ μL	261 U/L	Initial: 38.900 copies/mL 6 months after treatment: <20 copies/ml	Eltigravir Cobicistat Emtricitabin Tenofovir	DLBCL	Bcl-2 (-) Bcl-6 (+) c-myc (+) Mum1 (-) Ki67 85%	R-CHOP R-Bendamustin GDP Lenalidomid	IPI 1 Stage III ECOG 1
2	30	29	Initial: 169 cells/ μL 8 months after treatment: 292 cells/ μl	207 U/L	Initial: 1.076.402 copies/ml 6 months after treatment: 69 copies/ml	Eltigravir Cobicistat Emtricitabin Tenofovir Dolutegravir	DLBCL	Bcl-2 (-) Bcl-6 (+) c-myc (+) Mum1 (-) Ki67 95%	R-CHOP	IPI 0 Stage II ECOG 0
3	63	63	Initial: 407 cells/ μL 6 months after treatment: 371 cells/ μL	220 U/L	Initial: 2.390.000 copies/ml 6 months after treatment: 97 copies/ml	Eltigravir Cobicistat Emtricitabin Tenofovir	DLBCL	Bcl-2 (+) Bcl-6 (-) c-myc (-) Mum1 (+) Ki67 70%	R-CHOP	IPI:4 Stage IV ECOG 2
4	43	42	Initial: 21 cells/ μL 6 months after treatment: 32.16 cells/ μL	167 U/L	Initial: 89.200 copies/ml 6 months after treatment: 178 copies/ml	Eltigravir Cobicistat Emtricitabin Tenofovir	DLBCL	Bcl-2 (+) Bcl-6 (–) c-myc (+) Mum1 (+) Ki67 80% EBV (+)	R-CHOP	IPI 2 Stage II ECOG 1
5	33	33	Initial: 18 cells/ μL	325 U/L	Initial: Undetectable	Eltigravir Cobicistat Emtricitabin Tenofovir	DLBCL	Bcl-2 (-) Bcl-6 (-) c-myc (+)	R-CHOP	IPI 1 Stage I Bulky ECOG 1

### Case 4

A 43-year-old HIV (+) male patient presented with nasal congestion and fever to ear, nose and throat (ENT) specialist. Biopsy obtained from non-regressing lesion at left maxillary sinus revealed DLBCL while laboratory work-up showed positivity for EBV at that time. Past medical history of the patient was significant for eczema with exanthematous skin lesions and VDRL positivity treated with penicillin. Further evaluation demonstrated CD4 cell count of 21 cells/μL. Anti-viral therapy and 6 cycles of R-CHOP regimen were initiated. Following 6 cycles of chemotherapy complete metabolic response was achieved. Later on, patient presented to our clinic with nasal congestion during which biopsy obtained from right maxillary sinus revealed 10×7×2 mm soft irregular mass containing malignant kappa light chain plasma cell infiltration with CD38 positivity and lambda negativity considered as plasmacytoma. Bone marrow biopsy was obtained by the differential diagnosis of multiple myeloma demonstrated normocellular bone marrow without any atypia. Patient received radiotherapy for plasmacytoma. Follow-up of the patient has been uneventful since then.

### Case 5

A 33-year-old HIV (+) male patient with undetectable HIV RNA over 3 months was admitted to our clinic with bulky neck mass. MRI revealed an irregular 63×47×80 mm mass located posterior to sternocleidomastoid muscle after which PET scan demonstrated hypermetabolic profile of the cervical mass in addition to suspicious hypermetabolic lesions at spleen and adrenal gland. Patient described the absence of B-symptoms. Past medical history was significant for tonsillectomy and surgically-resected anal condyloma while family history was significant for hepatocellular carcinoma in father. Biopsy revealed germinal center DLBCL with positivity for Bcl-6 and c-myc, however, FISH analysis ruled out c-myc and Bcl-6 positivity. Patient received 2 cycles of R-CHOP regimen which was followed by 2 cycles of dose-adjusted R-EPOCH due to lack of metabolic response. Patient is still on the regimen.

## Discussion

HIV infection is a strong predisposing factor for malignant transformation, therefore, high risk patients presenting with advanced staged NHL or fast disease progression should be checked for concomitant HIV infection. Poor prognostic factors include low CD4 cell count (< 100 cells/ml), high LDH values, IV drug abuse, advanced stage and patients over age 35 (Yancheva *et al.*, 2018). In our case series; 3 of 5 patients had at least 2 poor prognostic factors, while all patients received ART (Table 1). In addition, our patients also suffer from comorbidities including eczema, hepatitis B virus (HBV) and EBV infections and syphilis. Only a single cohort including 1872 HIV-infected patients with malignancy rate of 2.6% has been performed in Turkish population (Aydin *et al.*, 2020). Therefore, our study is significant by demonstrating beneficial effects of HIV-related lymphoma cases in Turkey that received ART in addition to chemotherapy regimens.

Two commonly preferred chemotherapy regimens for HIV-related NHL cases are R-CHOP and dose-adjusted etoposide-vincristine-doxorubicin-oral prednisolone-dose-escalated cyclophosphamide (DA-EPOCH), while choice should be individualized. DA-EPOCH is recommended in CD20 (+) patients with plasmablastic histology or high growth fraction disease (Ki67>80%) unless CD4 cell count is below 50 cells/ml while none were present in our study population (Little *et al.*, 2003). Combination of rituximab and DA-EPOCH therapy should be considered in cases with poor prognostic factors, though, results of clinical trials are inconclusive (Dunleavy *et al.*, 2010; Sparano *et al.*, 2010).

Combination therapy with ART and chemotherapy leads to decline in HIV RNA levels and elevation at CD4 cell count in our cases. Declining viral load may be the underlying mechanism of beneficial effects of ART (Tirelli and Bernardi, 2001). No significant adverse effects of ART have been detected in our cases.

Another feature of HIV-associated lymphoma is the atypical presentation sites. Similarly, one of our cases presented with maxillary sinus involvement, while only limited number of cases with maxillary sinus lymphoma were present (Basavaraj *et al.*, 2016). Moreover, most HIV-associated lymphoma cases reported in maxillary sinus have been reported as plasmablastic lymphoma in contrast to our case presenting with DLBCL.

Despite studies indicating 1-year survival rate of 30% in HIV-associated lymphoma, individualized treatment modalities involving multidisciplinary approach have led to uneventful follow-up of all cases with HIV-associated lymphoma in our clinic for the last 3 years (Yancheva *et al.*, 2018). We assume that therapeutic outcomes of HIV (+) patients presenting with NHL might improve in the upcoming years with the improvements in ART options. Thus, we strongly suggest utilization of ART combined with chemotherapy in HIV-positive patients presenting with lymphoma to improve therapeutic outcomes.

**Abbreviations:**
HIV: -Human immunodeficiency virus
ART: -Antiretroviral therapy
HHV: -8-Human Herpes Virus-8
EBV: -Epstein Barr Virus
NHL: -Non-Hodgkin lymphoma
DLBCL: - Diffuse large B-cell lymphoma
CD: -Cluster of differentiation
R-CHOP: Rituximab-cyclophosphamide-doxorubicin-vincristine-prednisolone
GDP: -Gemcitabine-dexamethasone-cisplatin
R-DHAP: -Rituximab-cisplatin-cytosine arabinoside-dexamethasone
DA: -EPOCH-Dose-adjusted etoposide-vincristine-doxorubicin-oral prednisolone-dose-escalated cyclophosphamide
PET: -Positron emission tomography
FDG: -Fluorodeoxyglucose
CT: -Computed tomography
MRI: -Magnetic resonance imaging
SUVmax: -Maximal measuring standardized uptake value
FISH: -Fluorescence in situ hybridization
VDRL: -Venereal Disease Research Laboratory
ECOG: - The Eastern Cooperative Oncology Group
IPI: - The International Prognostic Index
Bcl: - B-cell lymphoma
Myc: -Myelocytoma
Mum1: - Multiple myeloma oncogene 1.

## References

[1] Aydin O. A, Gunduz A, Sargin F, Mete B, Karaosmanoglu H. K, Sevgi D. Y, Tabak F (2020). Prevalence and mortality of cancer among people living with HIV and AIDS patients:a large cohort study in Turkey. East Mediterr Health J.

[2]  Basavaraj A, Kadam M, &Kadam D. B (2016). Primary Maxillary Sinus Plasmablastic Lymphoma in HIV/AIDS. The Journal of the Association of Physicians of India.

[3] Dunleavy K, Little R. F, Pittaluga S, Grant N, Wayne A. S, Carrasquillo J. A, Wilson W. H (2010). The role of tumor histogenesis, FDG-PET, and short-course EPOCH with dose-dense rituximab (SC-EPOCH-RR) in HIV-associated diffuse large B-cell lymphoma. Blood.

[4] Grulich A. E, van Leeuwen M. T, Falster M. O, &Vajdic C. M (2007). Incidence of cancers in people with HIV/AIDS compared with immunosuppressed transplant recipients:a meta-analysis. Lancet (London, England).

[5] Haas A, Zimmermann K, &Oxenius A (2011). Antigen-dependent and -independent mechanisms of T and B cell hyperactivation during chronic HIV-1 infection. Journal of virology.

[6] Ji Y, &Lu H (2017). Malignancies in HIV-Infected and AIDS Patients. Advances in experimental medicine and biology.

[7] Little R. F, Pittaluga S, Grant N, Steinberg S. M, Kavlick M. F, Mitsuya H, Wilson W. H (2003). Highly effective treatment of acquired immunodeficiency syndrome-related lymphoma with dose-adjusted EPOCH:impact of antiretroviral therapy suspension and tumor biology. Blood.

[8] Opie J, Antel K, Koller A, &Novitzky N (2020). In the South African setting, HIV-associated Burkitt lymphoma is associated with frequent leukaemic presentation, complex cytogenetic karyotypes, and adverse clinical outcomes. Ann Hematol.

[9] Painschab M. S, Kasonkanji E, Zuze T, Kaimila B, Tomoka T, Nyasosela R, Gopal S (2019). Mature outcomes and prognostic indices in diffuse large B-cell lymphoma in Malawi:a prospective cohort. Br J Haematol.

[10] Rabkin C. S, &Yellin F (1994). Cancer incidence in a population with a high prevalence of infection with human immunodeficiency virus type 1. Journal of the National Cancer Institute.

[11] Smith C. J, Ryom L, Weber R, Morlat P, Pradier C, Reiss P, Lundgren J. D (2014). Trends in underlying causes of death in people with HIV from 1999 to 2011 (D:A:D):a multicohort collaboration. Lancet.

[12] Sparano J. A, Lee J. Y, Kaplan L. D, Levine A. M, Ramos J. C, Ambinder R. F, Consortium A. M (2010). Rituximab plus concurrent infusional EPOCH chemotherapy is highly effective in HIV-associated B-cell non-Hodgkin lymphoma. Blood.

[13] Tirelli U, &Bernardi D (2001). Impact of HAART on the clinical management of AIDS-related cancers. European journal of cancer (Oxford, England :1990).

[14] Yancheva N, Strashimirov D, Hrischev V, Tchervenyakova T, Nikolova M, &Aleksiev I (2018). Three cases of non-Hodgkin's lymphoma in HIV-infected Bulgarian patients. Le infezioni in medicina.

